# Genogroup IV and VI Canine Noroviruses Interact with Histo-Blood Group Antigens

**DOI:** 10.1128/JVI.01008-14

**Published:** 2014-09

**Authors:** Sarah Caddy, Adrien Breiman, Jacques le Pendu, Ian Goodfellow

**Affiliations:** a Division of Virology, Department of Pathology, University of Cambridge, Addenbrookes Hospital, Cambridge, United Kingdom; b Section of Virology, Faculty of Medicine, Imperial College London, St. Mary's Campus, London, United Kingdom; c INSERM, UMR892, and CNRS, UMR6299, Université de Nantes, Nantes, France

## Abstract

Human noroviruses (HuNV) are a significant cause of viral gastroenteritis in humans worldwide. HuNV attaches to cell surface carbohydrate structures known as histo-blood group antigens (HBGAs) prior to internalization, and HBGA polymorphism among human populations is closely linked to susceptibility to HuNV. Noroviruses are divided into 6 genogroups, with human strains grouped into genogroups I (GI), II, and IV. Canine norovirus (CNV) is a recently discovered pathogen in dogs, with strains classified into genogroups IV and VI. Whereas it is known that GI to GIII noroviruses bind to HBGAs and GV noroviruses recognize terminal sialic acid residues, the attachment factors for GIV and GVI noroviruses have not been reported. This study sought to determine the carbohydrate binding specificity of CNV and to compare it to the binding specificities of noroviruses from other genogroups. A panel of synthetic oligosaccharides were used to assess the binding specificity of CNV virus-like particles (VLPs) and identified α1,2-fucose as a key attachment factor. CNV VLP binding to canine saliva and tissue samples using enzyme-linked immunosorbent assays (ELISAs) and immunohistochemistry confirmed that α1,2-fucose-containing H and A antigens of the HBGA family were recognized by CNV. Phenotyping studies demonstrated expression of these antigens in a population of dogs. The virus-ligand interaction was further characterized using blockade studies, cell lines expressing HBGAs, and enzymatic removal of candidate carbohydrates from tissue sections. Recognition of HBGAs by CNV provides new insights into the evolution of noroviruses and raises concerns regarding the potential for zoonotic transmission of CNV to humans.

**IMPORTANCE** Infections with human norovirus cause acute gastroenteritis in millions of people each year worldwide. Noroviruses can also affect nonhuman species and are divided into 6 different groups based on their capsid sequences. Human noroviruses in genogroups I and II interact with histo-blood group antigen carbohydrates, bovine noroviruses (genogroup III) interact with alpha-galactosidase (α-Gal) carbohydrates, and murine norovirus (genogroup V) recognizes sialic acids. The canine-specific strains of norovirus are grouped into genogroups IV and VI, and this study is the first to characterize which carbohydrate structures they can recognize. Using canine norovirus virus-like particles, this work shows that representative genogroup IV and VI viruses can interact with histo-blood group antigens. The binding specificity of canine noroviruses is therefore very similar to that of the human norovirus strains classified into genogroups I and II. This raises interesting questions about the evolution of noroviruses and suggests it may be possible for canine norovirus to infect humans.

## INTRODUCTION

The family Caliciviridae is a diverse group of single-stranded RNA viruses that can infect a wide range of species. The virus family is divided into at least five genera, Lagovirus, Vesivirus, Nebovirus, Sapovirus, and Norovirus, and an additional four genera, Recovirus, Valovirus, Nacovirus, and Bavovirus (1–4), have been proposed. Caliciviruses can cause a variety of diseases in animals, including respiratory disease (feline calicivirus), hemorrhagic disease (rabbit hemorrhagic disease virus [RHDV]), and gastroenteritis (noroviruses and sapoviruses). In humans, noroviruses are a highly prevalent global pathogen, with up to 18% of all cases of acute gastroenteritis attributed to human norovirus (HuNV) in the United Kingdom (5) and 19 to 21 million cases occurring each year in the United States (6).

Many caliciviruses use carbohydrates as attachment factors to bind to cells prior to internalization. Murine norovirus (MNV) and feline calicivirus recognize forms of sialic acids (7, 8), bovine norovirus binds to alpha-galactosidase (α-Gal) (9), and it is also recognized that a number of caliciviruses bind to specific carbohydrates known as histo-blood group antigens (HBGAs). The Lagovirus RHDV was the first virus identified as using HBGAs as attachment factors (10), and this was soon followed by the demonstration that human Norwalk virus also uses these carbohydrates (11). Subsequent studies showed that the majority, if not all, of genogroup I (G1) and genogroup II human noroviruses recognize HBGAs. Most recently, the Tulane virus of the recently proposed genus Recovirus was also shown to bind these carbohydrate structures (12).

HBGAs are terminal structures of glycan chains expressed on the surfaces of specific cells. HBGAs are found on red blood cells in humans and great apes, as well as being located on epithelial cells of the gastrointestinal, genitourinary, and respiratory tracts in a wide variety of species. In addition, HBGAs can be secreted by these cells into bodily fluids, including saliva (13). The biosynthesis of HBGAs requires the stepwise addition of monosaccharide units onto glycan chains, a process performed by specific glycosyltransferases. HBGAs are derived from different types of precursor disaccharide structures; the type 1 precursor molecule is a galactose (Gal) joined to an *N*-acetylglucosamine (GlcNAc) via a β1,3 linkage. Type 2 is Galβ1,4GlcNAc. The type 3 precursor is a Galβ1,3GalNAc in α linkage to the subjacent structure (i.e., serine or threonine of the peptide chain), and the type 4 precursor is also a Galβ1,3GalNAc, but in β linkage to the subjacent structure. Conversion of these structures into the H antigen requires the action of α1,2-fucosyltransferases. On red blood cells and vascular endothelium, fucosyltransferase 1 (FUT1) is active, whereas in epithelial cells, FUT2 is active (11). Generation of the Lewis antigens also requires the addition of a fucose, but it is in an α1,3 or α1,4 configuration and requires the activity of either FUT3, FUT4, FUT5, FUT6, FUT7, or FUT9. Once the H antigens are formed, biosynthesis can continue by addition of either a GalNAc or a Gal in α1,3 linkage to give the A or B antigen, respectively. This step is catalyzed by the A or B enzymes, which are encoded by different alleles at the *ABO* locus (13).

Internalization of viral particles into cells occurs following HuNV attachment to HBGAs *in vitro*, and hence, the primary step for HuNV uptake into cells is believed to be HuNV binding to the HBGAs (11). However, it is worth noting that HBGA expression alone is not sufficient to enable infection of cells in culture (14), and at present, there is no cell culture system that allows study of the full replication cycle of HuNV. Approximately 20% of Caucasians do not express HBGAs on their epithelial cells due to mutations in the *FUT2* gene, producing a nonfunctional FUT2 enzyme (13). Prior to definitive clinical studies, it was hypothesized that these “nonsecretor” individuals would be resistant to Norwalk infection (11), and this was later confirmed in two study populations of 77 and 51 humans, respectively (15, 16).

Canine norovirus (CNV) is a recently discovered member of the genus Norovirus, first isolated from a dog with gastroenteritis in Italy in 2007 (17). Evidence for CNV infection has since been identified in Europe (18–20) and the Far East (21). CNV is believed to cause gastroenteritis in the canine host, though the virus has also been isolated from healthy dogs (19, 22). CNV strains have been assigned to genogroups IV and VI based on the capsid amino acid sequence. A seventh norovirus genogroup in which some CNV strains are classified has recently been proposed, but for this report these particular CNV strains maintain their initial GVI classification (23). A few rare HuNV genotypes have been grouped into genogroup IV (24, 25), alongside some of the CNV strains, although the majority of HuNV strains are classified in genogroups I and II.

It was predicted that CNV would use carbohydrate structures to attach to cells prior to infection in a similar manner to many other caliciviruses investigated to date. The aim of this study was to identify and characterize the interaction of the virus capsid with host cells. Expression of the target carbohydrate was then assessed in a population of dogs to examine the proportion of the canine population that may be susceptible to the virus.

## MATERIALS AND METHODS

### Ethics statement.

Human saliva samples were collected as part of a previous study (26) approved by the Nantes University Hospital Review Board (study no. BRD02/2-P), with informed written consent obtained from all saliva donors. Collection of canine saliva samples was a nonregulated procedure, and hence, ethical approval was not required. Canine gastrointestinal tissue samples were donated by a large pharmaceutical company. The six dogs had been bred for scientific research but were deemed unsuitable for the purpose and were humanely euthanized. The use of rats for antibody generation was approved by the national ethics review board of the French Ministry of Enseignement Supérieur et de la Recherche (project license number 01322.01). The animal care and use protocol adhered to European Directive number 2010/063 and to the national French regulation (Décret no. 2013-118 du 1er Février 2013 Relatif à la Protection des Animaux Utilisés à des Fins Scientifiques).

### Reagents and samples.

Anti-CNV polyclonal antibody was generated by serial inoculation of 2 rats with CNV virus-like particles (VLPs) (University of Nantes, animal experimentation core facility). Recognition of target VLPs using the antibody generated was confirmed by an enzyme-linked immunosorbent assay (ELISA).

Canine tissue samples were donated from six healthy 18-month-old female dogs (labeled A to F) humanely euthanized as surplus to industry research requirements. Sections of the gastrointestinal tract (1 cm^2^) were dissected from the duodenum, jejunum, ileum, cecum, and colon and placed into 90% ethanol fixative to best preserve the carbohydrate structures. Samples were then incubated at 4°C for 24 h prior to embedding in paraffin and sectioning. Additional 1-cm-long sections from the duodenum, jejunum, ileum, cecum, and colon were dissected, rinsed in phosphate-buffered saline (PBS), opened, and scraped into lysis buffer (GenElute Mammalian Total RNA Miniprep Kit [Sigma-Aldrich]) containing β-mercaptoethanol and guanidinium. The tissue scrapings were homogenized and boiled for 10 min prior to storage at −20°C. The mannose-binding lectin concanavalin A was used to confirm that comparable levels of carbohydrates were present in all scraping samples.

Canine saliva samples and DNA samples were collected from 23 dogs at Wood Green Animal Shelter, Huntingdon, United Kingdom (numbered 1 to 23), and a further 3 samples were collected from three of the dogs at a pharmacological research company in the United Kingdom (labeled D, E. and F). The dogs at the animal shelter were typically mixed breeds, whereas the research dogs were beagles. Sample collection was achieved using a children's swab (Salimetrics, Newmarket, United Kingdom), from which saliva and buccal cells (for DNA samples) were extracted. Human saliva samples were collected as part of a previous study (26).

### VLP production.

VLPs of three strains of CNV and human norovirus GI.1 (Norwalk virus) were generated using a previously described method (18). Recombinant baculoviruses containing VP1 protein from either a CNV strain or GI.1 were generated, and then, VLPs were produced by infection of Hi5 insect cells. VLPs were released from the infected Hi5 cells by three rounds of freeze-thawing and then clarified by removal of cellular debris (6,000 × *g*; 30 min) and baculovirus (14,000 × *g* for 30 min). The VLPs were partially purified through a 30% (wt/vol) sucrose cushion in TNC buffer (50 mM Tris-HCl, pH 7.4, 150 mM NaCl, 10 mM CaCl_2_) containing the protease inhibitor leupeptin at 150,000 × *g* for 2 h. The pelleted VLPs were resuspended in TNC and further purified by isopynic centrifugation in cesium chloride (150,000 × *g*; 18 h). The resultant VLP bands were collected by puncture, and the solution containing VLPs was dialyzed against PBS prior to quantification by bicinchoninic acid (BCA) protein assay (Thermo Scientific) and storage at −80°C.

### ELISA-based microtiter plate assays.

Nunc Maxisorp immunoplates were coated with synthetic oligosaccharides as polyacrylamide (PAA) and human serum albumin (HSA) conjugates (listed in Table 1) at 1 μg per well in 100 mM carbonate buffer, pH 9.6, by overnight incubation at 37°C in a humidified atmosphere. For assays using saliva and duodenal samples, immunoplates were coated with the same reagents at a 1:1,000 dilution under the same conditions.

**TABLE 1:** Neoglycoconjugates used to determine the carbohydrate specificities of CNV VLPs^*a*^

Name	Conjugate	Structure
α-*N*-Acetylgalactose	PAA	GalNAcα1
LacNAc	PAA	Galβ1-4GlcNAcβ1
Forsmann disaccharide	PAA	GalNAcα1-3GalNAcβ1
H type 1	HSA	Fucα1-2Galβ1-3GlcNAcβ1
H type 2	Both	Fucα1-2Galβ1-4GlcNAcβ1
H type 3	PAA	Fucα1-2Galβ1-3GalNAcα1
A heptasaccharide	HSA	GalNAcα1-3(Fucα1-2)Galβ1-3(Fucα1-4)GlcNAcβ1-3Galβ1-4Glcβ1
A trisaccharide	Both	GalNAcα1-3(Fucα1-2)Galβ1
A disaccharide	PAA	GalNAcα1-3Galβ1
A type 2b	PAA	GalNAcα1-3(Fucα1-2)Galβ1-4GlcNacβ1
Le^a^ (Lewis a)	PAA	Galβ1-3(Fucα1-4)GlcNAcβ1
Le^b^ (Lewis b)	Both	Fucα1-2Galβ1-3(Fucα1-4)GlcNAcβ1
Le^x^ (Lewis x)	PAA	Galβ1-4(Fucα1-3)GlcNAcβ1
Le^y^ (Lewis y)	Both	Fucα1-2Galβ1-4(Fucα1-3)GlcNAcβ1
3-Sulfo Le^x^	PAA	HSO_3_-3Galβ1-4(Fucα1-3)GlcNAcβ1
Sial Le^x^ (sialyl-Lewis x)	Both	NeuAcα2-3Galβ1-4 (Fucα1-3)GlcNAcβ1
Sial Le^a^ (sialyl-Lewis a)	Both	NeuAcα2-3Galβ1-4 (Fucα1-3)GlcNAcβ1
Sialyl LNF V (lacto *N*-fucopentose V)	HSA	Fucα1-2Galβ1-3(Neu5Acα2-6)GlcNAcβ1-3Galβ1-4Glc β1
Sialyl LNnT (lacto-*N*-neotetraose)	HSA	NeuAcα2-3Galβ1-4GlcNAcβ1-3Galβ1-4Glcβ1
6-Sulfo sialyl Le^x^	PAA	NeuAcα2-3Galβ1-3(Fucα1-4)(HSO_3_-6)GlcNAcβ1

a Twenty-six synthetic oligosaccharides, conjugated to either PAA or HSA, were used in ELISA-based assays to investigate the ability of CNV VLPs to bind carbohydrate structures. Ten of these synthetic oligosaccharides were only available conjugated to PAA, four were only available conjugated to HSA, and a further six oligosaccharide structures were available with either PAA or HSA conjugation.

After overnight coating, and in between all the subsequent incubation steps, the plates were washed 3 times with 0.05% Tween 20 in phosphate-buffered saline (PBS-T). The wells were then blocked with 5% milk in PBS-T for 1 h at 37°C before VLPs (10 μg/ml) in 5% milk in PBS-T were added to each well and incubated for 1 h at 37°C. Rat anti-CNV-VLP serum at 1/500 dilution in 5% milk in PBS-T was then added, and another 1-h incubation at 37°C took place. The final 1-h incubation at 37°C was with horseradish peroxidase (HRP)-conjugated anti-rat IgG (Uptima) at a 1/10,000 dilution in 5% milk in PBS-T. The enzyme signals were detected using TMB (3,3′,5,5′-tetramethylbenzidine) as the substrate (BD Bioscience, San Jose, CA), and the reaction was stopped with 1 N phosphoric acid. The optical density was read at 450 nm (OD_450_). To assess the effect of temperature on VLP binding to synthetic oligosaccharides, a limited number of experiments were repeated with all incubation steps at 4°C.

The saliva-phenotyping assay used the ELISA protocol detailed above, but with the following variations. Following the coating of immunoplates with saliva samples overnight, A antigen was detected using a mouse monoclonal anti-A antibody, 2A21, and B antigen was detected using a specific mouse monoclonal B49 antibody, a B-specific broadly reacting antibody, as previously described (27). Lewis antigen expression was investigated using mouse monoclonal antibodies 7-Le, 2.24LE, 3E1, and 12-4. H antigen expression was determined using HRP-conjugated Ulex europaeus I (Sigma-Aldrich, St. Louis, MO). Secondary HRP-conjugated anti-mouse (Uptima/Interchim, Montlucon, France) was used for A, B, and Lewis antigen detection.

1,2α-l-Fucosidase (Bifidobacterium bifidum) treatment of duodenal samples was performed by incubation at 37°C with 10 μg fucosidase in 100 mmol/liter sodium phosphate buffer, pH 6.5, for 1 h. Blocking the wells with 5% milk in PBS-T followed. The enzyme shows exquisite specificity for α1,2-linked fucose residues (28, 29).

Blocking of VLP binding with synthetic oligosaccharides was achieved by preincubating 10 μg/ml VLPs with 400 μg/ml oligosaccharides for 1 h at 37°C. The VLPs were then added to wells coated with duodenal samples, and the ELISA procedure followed as before.

### Sequencing of the canine α1,2-fucosyltransferase gene.

Canine DNA was extracted from the buccal epithelial cells collected with the Salimetric children's swab, using a commercial kit (GenElute; Sigma-Aldrich). Nucleotide sequence alignment of the human α1,2-fucosyltransferase (*FUT2*) gene and the predicted canine *Fut2* gene (GenBank accession number XM_005616863.1) enabled the design of primers to target the canine *Fut2* gene and allowed amplification of the entire gene for sequencing. The predicted canine *Fut2* gene has 87.8% identity with human *FUT2*. The primers IGUC0496 (TCCATCCYCCGAGCTAAC) and IGUC0497 (TCTGTTACTTGCCGCCCAAAGCAT) were used to amplify a region of DNA 1,020 bp long. Sequencing of PCR products was performed using primers IGUC0496, IGUC0497, and an additional primer (GGTACTCCTCCTCCATCCAGTCGT) by the University of Cambridge Biochemistry DNA-sequencing facility.

### Tissue samples and immunohistochemical analysis.

Tissue sections from the gastrointestinal tracts of six dogs were deparaffinized through baths of LMR-SOL (1-bromopropane,2-methylpropane-2-ol and acetonitrile), followed by rehydration with successive baths of 100, 90, 70, and 50% ethanol. Endogenous peroxidase activity was blocked with 0.3% hydrogen peroxide in PBS. Nonspecific binding was blocked with 3% bovine serum albumin (BSA) in PBS. HRP-conjugated U. europaeus I (Sigma-Aldrich, St. Louis, MO) at 0.8 μg/ml, anti-A monoclonal antibody 2A21, and anti-B monoclonal antibody B49 were used for binding to H antigen, A antigen, and B antigen, respectively. Lectins and antibodies were incubated with the tissue sections in 1% BSA in PBS at 4°C (lectin) or 37°C (antibodies) overnight. After three washes in PBS, a biotinylated anti-mouse antibody (Vector Laboratories, Burlingame, CA) diluted in 1% BSA in PBS was added to the assays with primary mouse antibodies. Sections were washed three times in PBS prior to addition of HRP-conjugated avidin D (Vector Laboratories, Burlingame, CA), also diluted in 1% BSA in PBS. Substrate was added to the slides (AEC kit; Vector Laboratories, Burlingame, CA), followed by Mayer's hematoxylin solution (Merck, Whitehouse Station, NJ) for contrast staining.

To assess the ability of VLPs to bind to tissue sections, the above-described protocol was adapted as follows. After sections were blocked with 3% BSA in PBS, 1 μg/ml VLPs was incubated with the sections overnight at room temperature. Anti-CNV primary antibody was then incubated with the tissue sections for 1 h at 37°C. After three washes in PBS, sections were incubated with secondary anti-rat biotinylated antibody (Vector Laboratories) for 1 h, and the remainder of the protocol was completed as previously described. Fucosidase treatment was performed on some sections after the initial blocking step in 3% BSA by incubation at 37°C with 10 μg fucosidase in 100 mmol/liter sodium phosphate buffer, pH 6.5, for a total of 18 h with renewal after 6 h.

### Flow cytometry analysis.

The binding of norovirus VLPs to cells *in vitro* was assessed using HT-29 (human colorectal adenocarcinoma cells), a cell line with well-characterized H antigen and A antigen expression. In addition, Chinese hamster ovary (CHO) cells transfected with rat α1,2-fucosyltransferase B cDNA, with or without cotransfection with the rat A enzyme cDNA or B enzyme cDNA, were utilized. The CHO cells were generated as part of a previous study (11). A total of 2.5 × 10^5^ viable cells were incubated with 10 μg/ml VLPs in PBS-0.1% BSA for 1 h at 4°C. After 3 washes with the same buffer, a 30-min incubation was performed with anti-VLP antibody. After washes, a third incubation was performed with biotinylated anti-rat secondary antibody under the same conditions. The final incubation step used streptavidin-phycoerythrin (BD Pharmingen). After final washes in PBS alone, fluorescence analysis was performed on a FACSCalibur (Becton, Dickinson, Rungis, France) using the CELLQuest program. Blocking of VLP binding with synthetic oligosaccharides was achieved by preincubating 10 μg/ml VLPs with 400 μg/ml oligosaccharides for 1 h at 37°C. Fucosidase treatment of cells was achieved by incubation at 37°C with 10 μg fucosidase in 100 mmol/liter sodium phosphate buffer, pH 6.5, for 1 h.

## RESULTS

### Recombinant CNV VLPs bind to synthetic neoglycoconjugates related to HBGAs.

**FIG 1:**
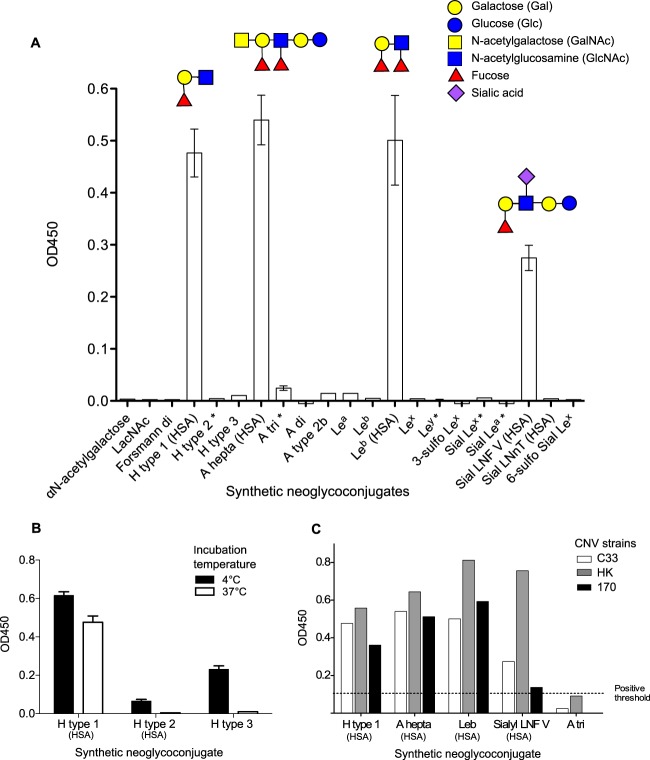
Binding of CNV VLPs to synthetic oligosaccharides. (A and B) CNV strain C33 VLPs were incubated with a panel of 26 neoglycoconjugates at 37°C (A) and a panel of 5 neoglycoconjugates at 4°C (B) immobilized on immunoplates to determine binding ability. Bound VLPs were detected using an anti-CNV antibody. Synthetic oligosaccharides were conjugated to either HSA or PAA (unlabeled). An asterisk indicates that the oligosaccharide was available for testing attached to both types of conjugate, and the mean OD_450_ for both is presented. VLP binding to Lewis b was significantly different for each conjugate, and hence, each is shown independently. Schematic structures of the neoglyconjugates recognized by CNV VLPs are presented above the associated bars on the chart. (C) Neoglycoconjugates shown to bind CNV strain C33 were also incubated with two additional CNV strain VLPs (170 and HK) at 37°C. The error bars represent standard errors.

It was hypothesized that CNV would be able to bind to cell surface carbohydrates, based on previous studies on attachment factors for other caliciviruses. Feline calicivirus; rabbit hemorrhagic disease virus; and human, bovine, and murine noroviruses can all recognize cell surface carbohydrates (7–11). To commence investigations, a panel of immobilized neoglycoconjugates were used to assess the ability of CNV to attach to them *in vitro*. VLPs of three different strains of CNV were incubated with a panel of immobilized neoglycoconjugates at 37°C to identify likely binding partners. The three CNV strains selected for this study are listed in Table 2. All strains were isolated in 2007, and the amino acid identities between strains were 58.4% to 60.6%. VLPs of CNV strain C33 were first tested against an extensive panel of neoglycoconjugates attached to either PAA or HSA (Fig. 1A). Four different carbohydrate structures to which VLPs could bind were identified. They were H type 1, A heptasaccharide (A hepta), Lewis b, and lacto-*N*-fucopentose. Each of these neoglycoconjugates incorporates the H type 1 motif Fucα1-2Galβ1-3GlcNAcβ1-R1 (the structures are shown in Fig. 1A), suggesting that these three carbohydrate moieties in the specific H type 1 configuration are important for CNV binding. This is in agreement with the finding that, although A heptasaccharide could bind to CNV VLPs, the closely related A disaccharide (A di) and A trisaccharide (A tri) neoglycoconjugates could not. A di and A tri neoglycoconjugates incorporate a GalNAc moiety, which differentiates A antigen from H antigen, but they lack GlcNAc, which is one of the three moieties that constitute the H antigen.

**TABLE 2:** CNV strains from which VLPs were produced for use in this study

CNV strain	GenBank accession number	Abbreviation	Genogroup
GIV.2/Bari/170/07-4/ITA	EU224456.1	170	IV
C33/Viseu/2007/PRT	GQ443611.1	C33	VI
GVI.1/HKU_Ca035F/2007/HKG	FJ692501.1	HK	VI

All four neoglycoconjugates shown to bind VLPs were attached to HSA, although there were six additional HSA-conjugated oligosaccharides that did not bind to the VLPs. The conjugate molecule is suspected to have some effect on the accessibility of the oligosaccharide, as Lewis b conjugated to PAA was not able to bind to the VLPs. This is evidently a limitation of the synthetic neoglyconjugate binding assay, and subsequent experiments using clinical samples were deemed essential to investigate these binding preferences further.

Temperature has previously been shown to affect the ability for norovirus VLP binding to H-type carbohydrates (30), so to investigate this possibility, synthetic oligosaccharides were incubated with VLPs at 4°C, and the effect on VLP binding was examined. Immunoplates were coated with H type 1 (HSA), H type 2 (HSA), and H type 3 (PAA), and C33 CNV VLPs were added for a 1-h incubation at 4°C. The results presented in Fig. 1B show that H type 2 and H type 3 oligosaccharides were able to weakly bind CNV VLPs at 4°C but showed no ability to bind CNV VLPs at 37°C.

Given the apparent specificity of CNV strain C33 VLP binding to neoglycoconjugates, it was predicted that the other two CNV strains available for this study would also behave in a similar manner. To investigate this, strains 170 and HK were incubated with the 4 neoglycoconjugates shown to bind strain C33 plus a negative control (A tri). The results shown in Fig. 1D confirmed that at 37°C, all 3 strains of CNV were able to recognize H type 1, A heptasaccharide, Lewis b, and lacto-*N*-fucopentose but could not bind to A tri (which lacks the GlcNAc of the type 1 precursor).

### Major capsid protein (VP1) sequence analysis of different noroviruses.

**FIG 2:**
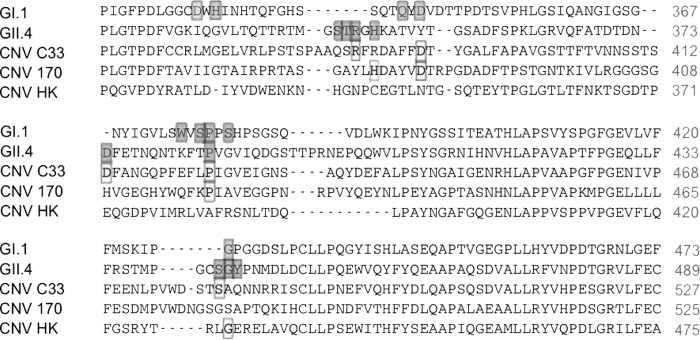
Alignment of GI.1, GII.4, and CNV major capsid protein sequences. Major capsid protein sequences of representative GI.1 (GenBank AAA59229.1) and GII.4 (GenBank ADR78268.1) human noroviruses and the three CNV strains used in this study were aligned using ClustalW. The 8 (GI.1) and 7 (GII.4) residues implicated in HBGA binding from crystallographic studies are shaded. Amino acid residues in the three different CNV strain major capsid proteins that are identical to the key HBGA-binding residues of GI.1 or GII.4 are boxed.

Genogroup I and genogroup II human noroviruses are known to bind to HBGAs, and following the discovery that CNV VLPs were able to bind to synthetic HBGA carbohydrates *in vitro*, comparison of the major capsid protein amino acid sequences was performed. The major capsid proteins of a representative GI.1 Norwalk virus strain (GenBank accession no. AAA59229.1) and a GII.4 strain (norovirus Hu/II.4/2201480/HK/2010; GenBank accession no. ADR78268.1) were aligned with the three CNV strains (Table 2) using ClustalW. Figure 2 illustrates only the regions of capsid sequence alignments that incorporate the amino acid residues shown to interact with HBGAs in previous crystallography studies of GI.1 and GII.4 noroviruses (31–33). Only very limited similarities were identified between the GI.1 or GII.4 norovirus HBGA-binding amino acids and the capsid sequences of the three CNV strains. This suggests that different mechanisms of carbohydrate binding between CNV and the GI and GII human noroviruses exist.

### Dogs express A, H, and Lewis antigens in their gastrointestinal tracts.

**FIG 3:**
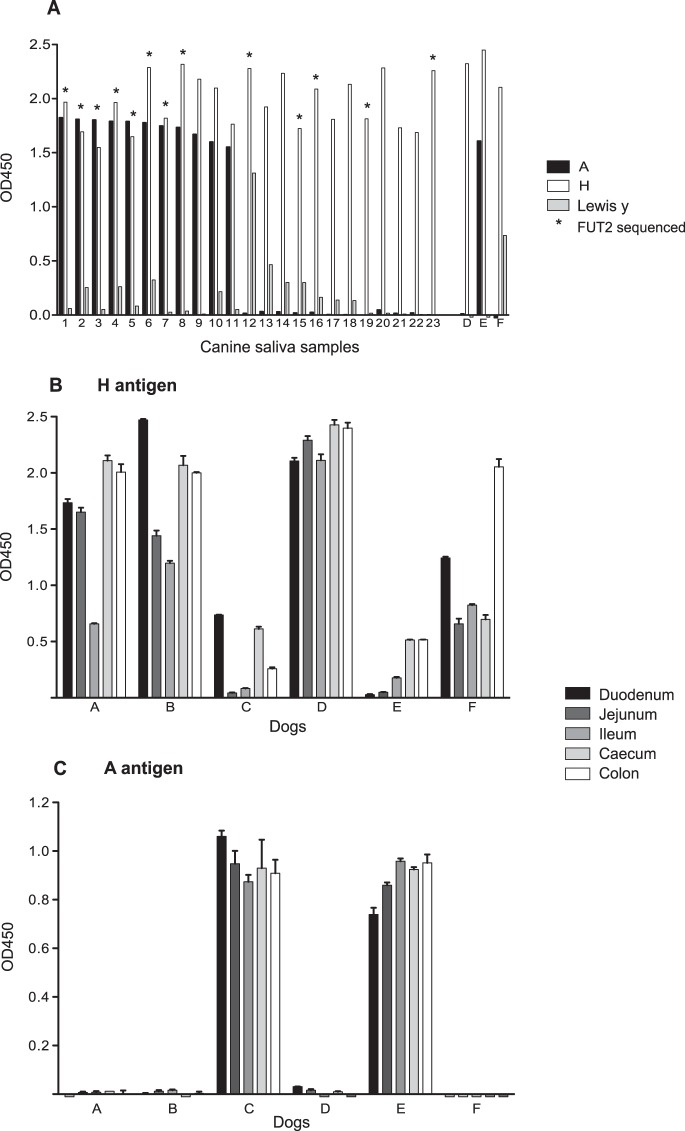
Phenotyping of canine saliva and gastrointestinal samples using ELISA-based assays. (A) Twenty-six canine saliva samples were analyzed to determine the expression of H antigen, A antigen, and Lewis y antigen. Saliva samples 1 to 23 were collected from kenneled dogs, whereas samples D, E, and F were collected from research dogs, from which tissues were also collected. The *Fut2* gene was sequenced for 14 dogs, identified by asterisks, in addition to dog B, from which saliva was not available. (B and C) Phenotyping for H antigen (B) and A antigen (C) was also performed for tissue scrapings from the duodenum, jejunum, ileum, cecum, and colon of the six dogs A to F. The error bars represent standard errors.

To determine if dogs express the carbohydrates identified as potential CNV attachment factors by the synthetic oligosaccharide assay, saliva samples from 26 dogs (1 to 23 and D to F) and intestinal scrapings from 6 dogs (A to F) were phenotyped using ELISA-based assays. H antigen carbohydrate was present in the saliva and intestinal tissue of all the dogs tested (Fig. 3A). These data were verified by sequencing the complete *Fut2* gene of 14 of these dogs (identified by asterisks in Fig. 3A). The canine *Fut2* gene showed high conservation of the nucleotide sequence between samples, with no evidence of polymorphisms that would result in an inactive transcript. A single noncoding nucleotide polymorphism (C777G) was identified in one dog.

Expression of H antigen along the length of the gastrointestinal tract was examined using samples collected from the duodenum, jejunum, ileum, cecum, and colon. Concanavalin A was used to confirm that the amounts of carbohydrate present in all the duodenal scrapings were comparable (data not shown). Figure 3B shows that H antigen expression in the intestines varies between dogs, but it is apparent that this inversely correlates with A antigen expression (Fig. 3C). Within the gastrointestinal tract of each dog, H antigen expression is relatively constant from the duodenum to the colon. This is in contrast to the H antigen expression patterns reported in humans and in cattle, where expression of the H antigen diminishes in the distal parts of the gastrointestinal tract (9, 34).

A antigen was shown to be present in 12/26 (46.2%) dogs, whereas no dogs were B antigen positive. A expression in saliva correlated with A expression in gastrointestinal secretions, as demonstrated by phenotyping of saliva samples (D, E, and F) obtained from three dogs from which gastrointestinal tissues were also available. Of the dogs from which tissues were obtained, two were identified as being A antigen positive (C and E), and A antigen expression throughout the length of the gastrointestinal tract in the two animals was relatively constant (Fig. 3C). The A-positive dogs had comparatively reduced expression of H antigen, as detected by *Ulex* binding, which is apparent in Fig. 3B. This is understood to be due to the ability of the A antigen to mask the H antigen, thus preventing detection by *Ulex* lectin binding (27).

Lewis a, Lewis b, and Lewis x were not detectable in canine saliva or canine gastrointestinal scrapings (data not shown). A lack of expression of Lewis a and Lewis x antigens was expected based on previous data that had already confirmed H antigen expression and therefore secretor status in every dog studied (Fig. 3A). Lewis y was detectable in 12/26 (46.2%) dogs, although Lewis y antigen expression was not linked to A antigen expression; dogs could express both, either, or neither antigen (Fig. 3A). Lewis a and b are derived from α1,3-fucosyltransferase activity on the type 1 precursor and H type 1, respectively, whereas Lewis x and y are derived from α1,3-fucosyltransferase activity on the type 2 precursor and H type 2. Among the Lewis y-positive dogs, Lewis y expression varied significantly, unlike the all-or-nothing expression of A antigen.

### CNV VLPs bind to intestinal tissues of A antigen-positive and -negative dogs.

**FIG 4:**
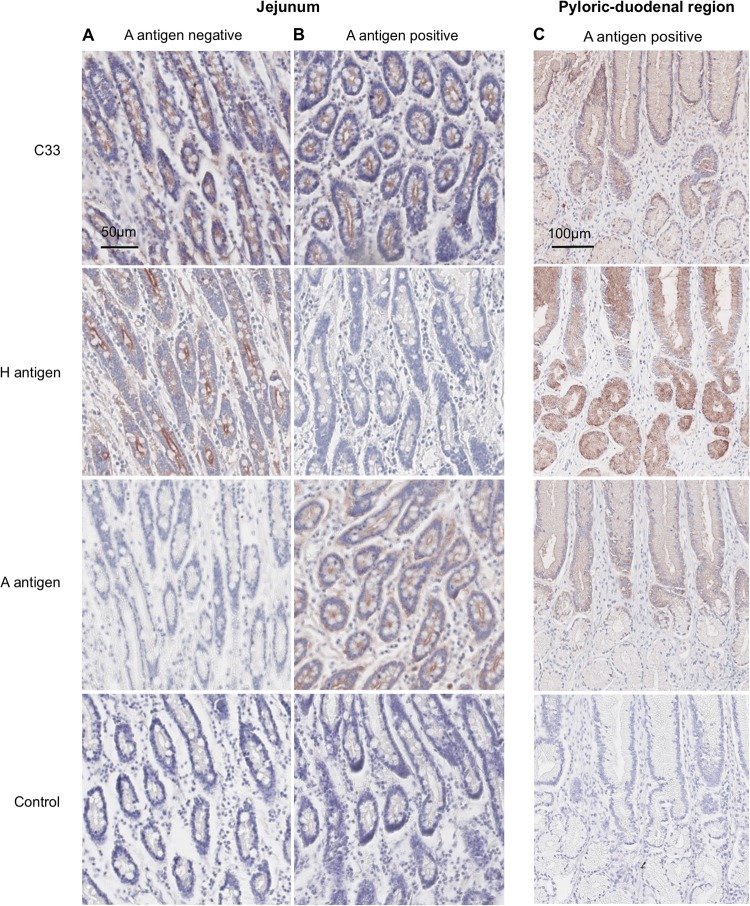
Immunohistochemical analysis of CNV VLPs binding to canine intestinal tissue sections. VLPs were incubated with tissue sections overnight, and binding was detected using anti-CNV antibody and biotinylated secondary antibody. HBGA expression was determined using anti-A antigen antibody and *Ulex* lectin. Binding of either VLPs or antibodies/lectin is indicated by the presence of red signal. (A) Binding of CNV strain C33 to jejunal tissue from an A antigen-negative dog. (B) C33 binding to jejunal tissue from an A-positive dog. (C) Binding of C33 to tissue from the pyloric duodenal region of the intestine of an A-positive dog.

Following identification of putative attachment factors for CNV and the confirmation that expression of most of these factors is present in the canine intestinal tract, we examined the ability of CNV VLPs to bind to canine samples. Immunohistochemistry was used to confirm that CNV VLPs could bind to canine intestinal sections and also to investigate the pattern of VLP binding and compare it to carbohydrate expression. Based on the data obtained using the panel of synthetic neoglycoconjugates, it was hypothesized that CNV binding would follow H antigen and A antigen expression in the tissues. Tissue sections from the length of the canine intestinal tract were incubated with CNV VLPs, and their binding was detected using primary and secondary antibodies. VLPs from the three representative strains of CNV (C33, 170, and HK) were incubated with tissue sections separately, and it was shown that all the strains were able to bind to the intestinal tissue sections used in this study (Fig. 4 and data not shown). Figure 4 shows only data obtained using the CNV strain C33 VLP binding to tissue sections, as it is representative of the two additional strains (data not shown).

To determine the presence or absence of H antigen and A antigen in the tissue sections used for the VLP binding, sections were incubated with *Ulex* and anti-A antigen antibodies, respectively, and immunohistochemistry was performed. Both A-positive and A-negative dogs were compared, as shown in Fig. 4A and B. H antigen expression was not detectable in the A-positive dogs, in agreement with the ELISA phenotyping data. Figure 4 demonstrates that CNV VLPs can bind to both A antigen-positive and A antigen-negative dogs and that expression of both of these carbohydrates follows a pattern comparable to that of CNV VLP binding. In particular, VLP binding closely follows the pattern of A expression when examining deeper tissues of the pyloric duodenal region (Fig. 4C). VLP binding was concentrated at the mucosal surfaces of intestinal villi, with no binding observed in deeper tissues. While this does not prove a direct association between CNV VLP and HBGA binding, it does add support to the initial data.

### CNV VLP binding ability shows variation related to the carbohydrate expression pattern.

The phenotyping results demonstrated that different dogs exhibit variation in carbohydrate expression comparable to that of most species studied. To investigate whether this affected the ability of CNV VLPs to bind to tissues, further assays were required to extend the findings provided by the initial immunohistochemical studies.

**FIG 5:**
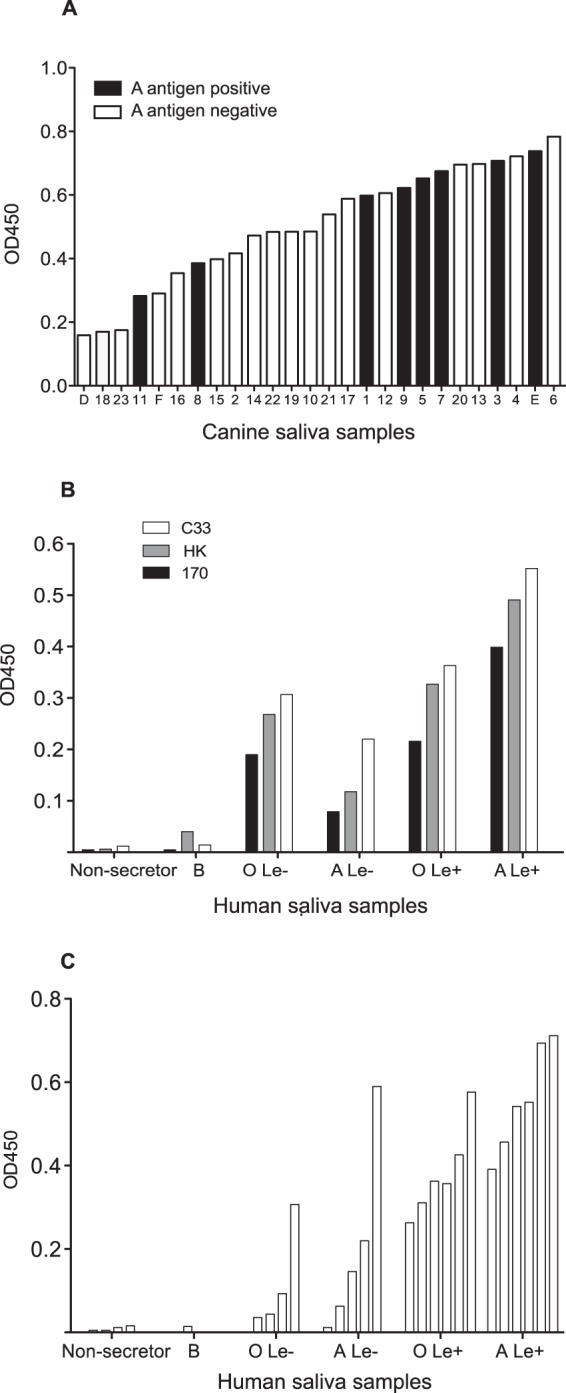
CNV VLP binding to canine and human saliva samples. (A) ELISA plates were coated with 26 canine saliva samples with characterized phenotypes (A antigen and Lewis), and the abilities of CNV VLPs (strain C33) to bind were assessed. The samples are ordered according to their CNV VLP binding abilities (low to high). Lewis antigen expression is not shown. (B) Six human saliva samples, each with a different ABO and Lewis phenotype, were used to assess binding of CNV VLPs from the three CNV strains available. (C) CNV strain C33 was next selected as the representative strain to analyze binding to a wider panel of 26 human saliva samples. Le, Lewis antigen.

CNV VLPs were incubated with 26 canine saliva samples in an ELISA-based assay and shown to bind to all of the samples (Fig. 5A). CNV strain C33 was selected as a representative strain for these experiments, as its binding was more consistent. The OD_450_ value for the binding of VLPs to each saliva sample was variable, with a range from 0.076 to 0.784. This variation was compared to the HBGA phenotype identified in each dog to see if any correlation between binding and expression of A antigen or Lewis antigen could be established. Although no statistically significant patterns were identified, a general trend was seen with regard to samples that most weakly and most strongly bound the CNV VLPs. Of the 3 saliva samples that bound the VLPs most weakly (OD_450_ < 0.2), none were A antigen positive. In contrast, all 4 of the saliva samples that bound to VLPs with an OD_450_ of >0.7 were A antigen positive. A significant relationship between Lewis antigen expression and VLP binding was not identified (data not shown).

As the carbohydrate repertoire of canine saliva samples has, as far as we are aware, not been characterized, it is likely that carbohydrates are present that were not detected during the phenotyping studies (Fig. 3) but that contribute to the variation in CNV VLP binding. In contrast to the paucity of data on carbohydrate expression in canine saliva, carbohydrate expression in human saliva is much better characterized. Therefore, it was anticipated that the use of a comprehensively phenotyped human saliva panel would provide more conclusive results regarding CNV VLP binding ability. To this end, 26 human saliva samples representing the six main groups of HBGA expression patterns known (O Lewis negative, O Lewis positive, A Lewis negative, A Lewis positive, B expression, and nonsecretor) were used in ELISA-based assays to quantitatively study CNV VLP binding. All three CNV strains available were first incubated with a single sample from each of the six HBGA groups, and identical binding patterns were identified for each strain (Fig. 5B). CNV strain C33 was then selected as a representative strain for the complete 26-sample panel (Fig. 5C).

Figures 5B and C showed that human saliva samples from nonsecretor individuals were not recognized by CNV VLPs. Nonsecretors do not have a functional *FUT2* gene and hence cannot express HBGAs on the surfaces of epithelial cells. This confirms that expression of HBGAs is essential for CNV binding. The single B antigen-positive saliva sample available in this panel also did not bind CNV VLP. B antigen expression was confirmed using the anti-B antibody B49. The structure of the B antigen (Galα1-3[Fucα1-3]Galβ1-GlcNAcβ1-R1) must therefore preclude binding, showing that the terminal galactose cannot be accommodated by the CNV VLP.

Saliva samples containing the O antigen (H antigen) and A antigen, with or without the presence of Lewis antigen, could all bind CNV VLPs to varying degrees. Excluding nonsecretor and B antigen-positive samples, the differences between the OD_450_ values for the saliva phenotypes were statistically significant (*P* = 0.013; one-way analysis of variance). Figure 5C demonstrates that human saliva samples have an increased ability to bind CNV VLPs if they contain A antigen and/or Lewis antigen instead of O (H) antigen alone.

**FIG 6:**
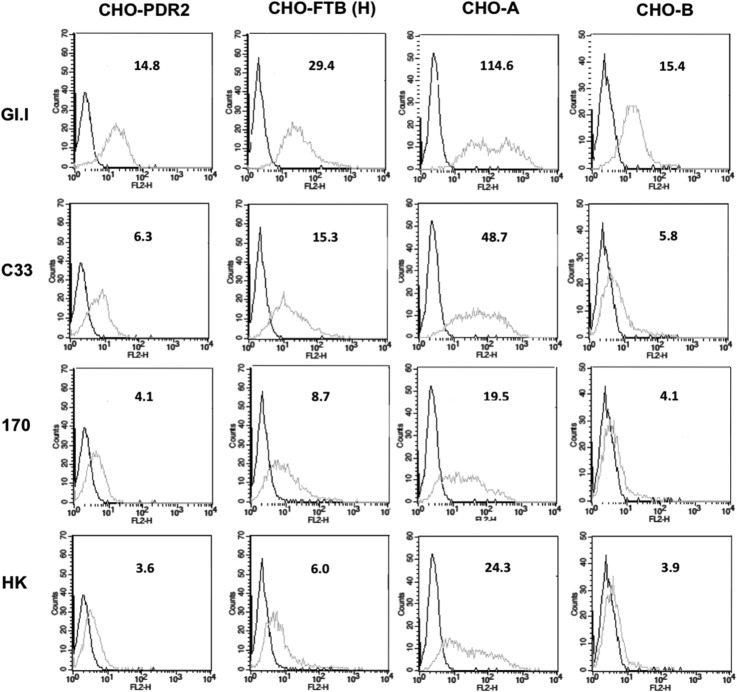
Binding of CNV and GI.1 VLPs to CHO cells transfected with glycosyltransferases. CHO cells were transfected with rat α1,2-fucosyltransferase B (FTB) cDNA to induce H antigen expression [CHO-FTB (H)] and were cotransfected with FTB and A enzyme to express A antigen (CHO-A) or transfected with FTB and B enzyme to express B antigen (CHO-B). CHO cells transfected with the empty PDR2 vector were used as control cells not expressing HBGAs. Binding of the three CNV strain VLPs (C33, 170, and HK) and GI.1 VLPs to the different cell lines was assessed using FACS. The black lines represent signal in the absence of VLPs but in the presence of the primary and secondary antibodies. The gray lines represent VLP binding. The number above each histogram is the mean fluorescence intensity (MFI) (geometric mean).

To extend these findings further, binding of VLPs to CHO cell lines transfected with glycosyltransferases was studied using fluorescence-activated cell sorting (FACS) (Fig. 6). CHO cells do not express α1,2-fucosyltransferase activity and hence are devoid of ABH antigens (11). Transfection of fucosyltransferase and A or B enzymes enabled the precise control of ABH antigen expression. Assessment of human norovirus G1.I VLPs and three strains of CNV VLPs binding to transfected and untransfected cells was achieved using FACS. This demonstrated that the GI.1 and CNV VLPs were able to bind CHO cells expressing H and A antigens, with a preference for A, but binding to cells expressing B antigen or no HBGAs at all was substantially less. It is interesting that HBGA expression of the transfected CHO cells was primarily type 2 structures (unpublished data). Binding of CNV strain C33 VLPs to type 2 structures at 37°C is contrary to the results obtained with the synthetic oligosaccharide assay (Fig. 1A), although it does agree with the data from ELISAs with 4°C incubation steps (Fig. 1B). This indicates that the CNV C33 H antigen specificity is not absolutely dependent on the β1,3 linkage of the Gal-GlcNAc moiety (H type 1) and can accommodate a β1,4 linkage (H type 2).

### Synthetic neoglycoconjugates incubated with VLPs can block binding to canine samples.

**FIG 7:**
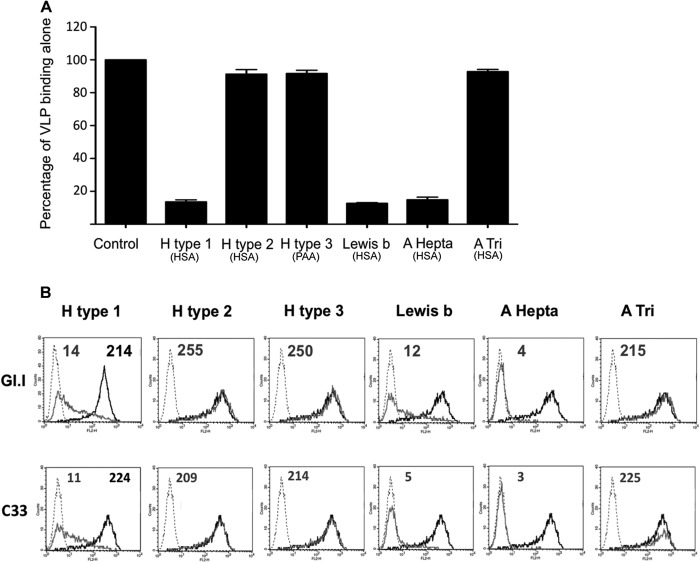
Blocking CNV binding to canine samples using synthetic neoglycoconjugates. Neoglycoconjugates were incubated with CNV VLPs for 1 h at 37°C prior to the VLPs being added to either duodenal scrapings in an ELISA-based assay (A) or human HT29 cells in suspension for FACS (B). Synthetic oligosaccharides were conjugated to either HSA or PAA, as indicated beneath each bar. The same conjugates were used in the FACS experiments, where the binding of both CNV strain C33 VLPs and human norovirus GI.1 VLPs was studied. The dashed lines represent signal in the absence of VLPs but in the presence of the primary and secondary antibodies. The black lines represent VLP binding when VLPs were preincubated with PBS only. The gray lines represent the binding of VLPs to cells after they had been preincubated with different synthetic oligosaccharides. The number in gray above each histogram is the MFI (geometric mean) of all cells when VLPs were preincubated with neoglycoconjugate. The second number, in black, for the H type 1 histograms is the MFI of all cells when VLPs were preincubated with PBS only. The error bars represent standard errors.

To further characterize the canine carbohydrates involved in recognition of CNV VLPs, a series of blocking studies were performed. The initial ELISA-based synthetic neoglycoconjugate assays had identified H antigen, A antigen, and Lewis b antigen as likely binding partners of the CNV VLPs. To investigate the roles of these carbohydrates further, CNV strain C33 VLPs were preincubated with each synthetic neoglycoconjugate prior to addition of the VLPs to either immunoplates coated with duodenal scrapings (Fig. 7A), cells in tissue culture (Fig. 7B), or tissue sections (data not shown). Preincubation of VLPs with H type 1, A hepta (A Lewis b), and Lewis b neoglycoconjugates significantly reduced VLP binding to canine duodenal scrapings (Fig. 7A) and to HBGA-expressing HT-29 cells in tissue culture (Fig. 7B). In contrast, when VLPs were preincubated with H type 2, H type 3, and A tri (a truncated form of A antigen lacking GlcNAc) at 37°C, no reduction in binding to canine samples was observed. Human norovirus G1.I VLPs were included in the FACS studies to provide a comparison set of data for a well-characterized norovirus-carbohydrate binding pattern. Overall, these blockade studies provided further evidence of the importance of H type 1, A antigen, and Lewis antigen in CNV binding.

### Enzymatic removal of specific intestinal carbohydrates reduces CNV binding.

**FIG 8:**
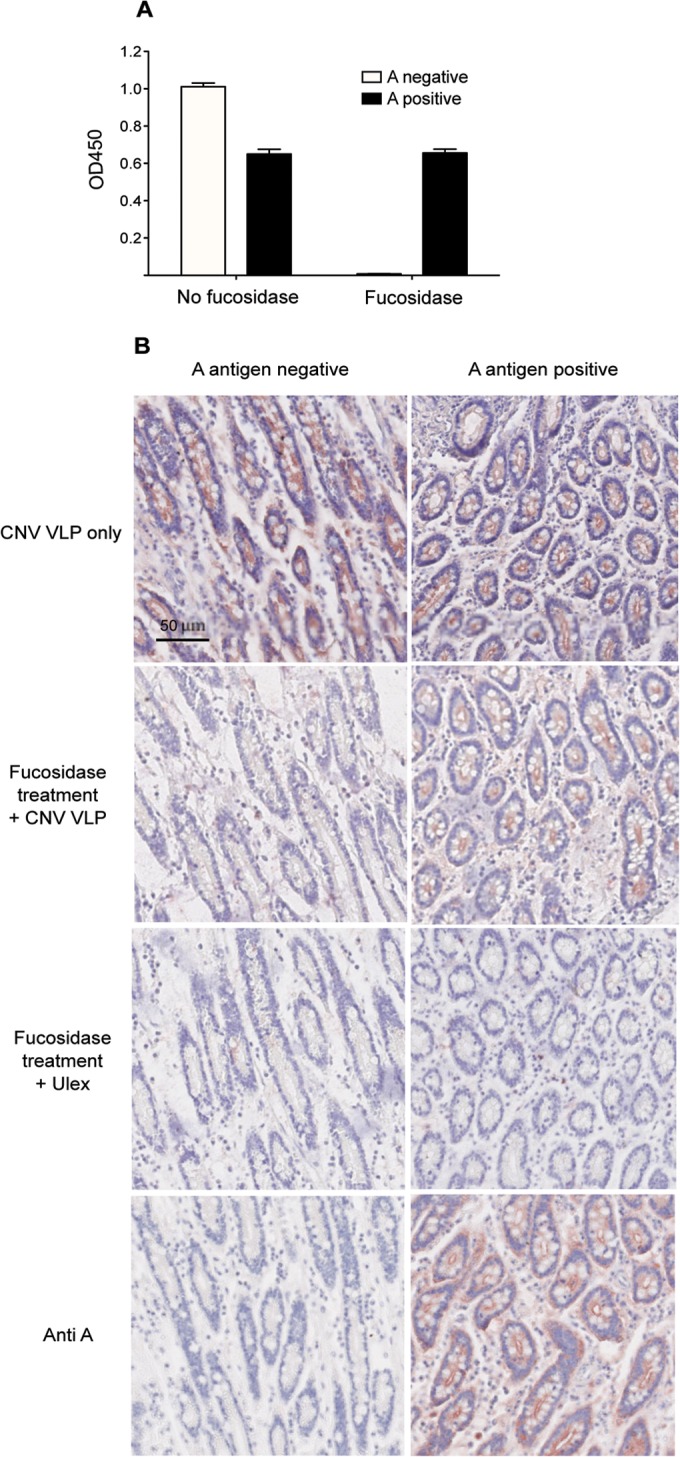
Enzymatic treatment of canine samples reduces CNV VLP binding. Duodenal scrapings and intestinal tissue sections from an A antigen-positive and an A antigen-negative dog were treated with 1,2-α-fucosidase or a control for 1 h (scrapings) or 18 h (tissue sections) at 37°C. The abilities of CNV VLPs (strain C33) to bind to the scrapings or tissue sections were assessed using an ELISA-based assay (A) and immunohistochemistry (B), respectively. Confirmation that the 1,2-α-fucosidase removed H antigens was achieved by incubating treated tissue sections with *Ulex* (Anti-H). The error bars represent standard errors.

Carbohydrate moiety-specific enzymes were used to cleave off HBGAs shown to play a role in CNV attachment to the cell surface. 1,2α-Fucosidase was incubated with duodenal scrapings, tissue sections, and cells in tissue culture to remove the fucosidase of the H antigen (Fig. 8). Fucosidase treatment was shown to completely abolish CNV VLP binding in A antigen-negative dogs. In dogs expressing A antigen, however, fucosidase treatment had no effect on CNV binding to duodenal scrapings and only a moderate effect on binding to tissue sections. Similarly, in A antigen-expressing cells used for FACS, no significant change in CNV VLP binding was observed after fucosidase treatment (data not shown). From this it can be concluded that in A-negative dogs, the H antigen is essential for CNV binding. Fucosidase treatment of A-positive dogs does not result in a change in the binding pattern because it is understood that the A antigen masks the H antigen-blocking fucosidase action (27); thus, A antigen will still be present and sufficient to mediate CNV VLP binding.

## DISCUSSION

This study demonstrates that CNV capsid can attach to the H antigen of the histo-blood group antigen carbohydrates expressed on the surfaces of epithelial cells. The interaction between the CNV capsid and the H antigen can accommodate the A antigen (addition of *N*-acetylgalactosamine) and the Lewis antigen (addition of a α1–3/1-4 fucose) but not the B antigen (addition of galactose). These conclusions have been drawn from initial assays using synthetic neoglycoconjugates and CNV VLP binding to intestinal sections, saliva, duodenal scrapings, and cells *in vitro*. Blocking assays and enzymatic removal of identified carbohydrates were then used to confirm these findings. While the merits of the individual assays may be limited (for example, it is predicted that the conjugate molecules of the neoglyconjugates can affect VLP binding and may not be an accurate reflection of the situation *in vivo*), the multiple lines of evidence for HBGA binding to CNV support this overall conclusion.

The discovery that CNV binds to HBGAs was unexpected with regard to the amino acid sequence of the CNV strains. Compared to the Norwalk virus capsid amino acid sequence, there was almost no similarity in the amino acids shown to interact with HBGAs (Asp327, His329, Gln342, Asp344, Trp375, Ser377, Pro378, and Ser380) (31, 33). When CNV major capsid protein sequences were compared to those of a GII.4 norovirus, a comparable lack of amino acid identity was observed in the key HBGA-binding residues (Ser343, Thr344, Arg345, Asp374, Ser441, Gly442, and Tyr443 [32]). The difference between the amino acid and HBGA interaction profiles for the genogroup I and genogroup II noroviruses indicates convergent evolution of the two genogroups has occurred (35). CNVs have been classified into GIV and GVI genogroups, genetically distant from the GI and GII human norovirus strains studied to date. These are the first data to suggest that norovirus recognition of HBGAs may have evolved in at least three different lineages. An alternative explanation for this finding is the existence of a distant ancestor of all norovirus strains that evolved the ability to recognize the HBGA carbohydrates that are conserved among many species. To begin establishing which proposal is correct, crystallographic studies of the CNV major capsid protein will be essential to enable identification of amino acids involved in HBGA recognition.

A limited number of human norovirus strains have been classified into genogroup IV (24, 25), alongside several of the CNV strains. The prototype CNV strain was shown to have 69% amino acid identity to human strains (17), and hence, CNV has been designated a GIV.2 norovirus while the human strains are GIV.1. The carbohydrate binding specificities of the human GIV strains have not been studied. The results from this work suggest that the human GIV noroviruses may also recognize HBGAs, continuing the binding specificity identified for previously investigated human noroviruses. To our knowledge, no human GIV VLPs have been generated, and hence, production and study of VLPs representative of the human GIV strains would be an interesting future line of investigation.

Identification of H, A, and Lewis antigens as the key ligands for CNV correlates well with the canine carbohydrate expression phenotypes identified in this study. All the dogs tested were positive for the H antigen, and no inactivating mutations were identified in the canine *Fut2* genes sequenced. This is in contrast to *FUT2* polymorphism in humans, where approximately 1 in 5 Caucasians have inactivating *FUT2* polymorphisms, resulting in the nonsecretor phenotype. The polymorphism G428A is responsible for this phenotype in over 95% of nonsecretors of European and African decent, and the A385T polymorphism is predominant in Asian nonsecretors (36, 37). In comparison, the single polymorphism identified in canine *Fut2* was a noncoding mutation, predicted to be a random change as opposed to a fixed polymorphism. Overall, the phenotyping and genotyping data obtained indicated that nonsecretor individuals were absent from this canine population. This could be a reflection of the lack of evolutionary pressure to induce resistance alleles.

No dogs were positive for the B antigen, in agreement with two previous reports examining carbohydrate expression in canine intestines (38, 39). A total of 12/26 dogs were positive for the A antigen, a proportion comparable to that in a 1982 study that analyzed fucolipid expression in the intestines of 37 dogs and identified 17 (46%) as A antigen positive (40). The same study identified Lewis b antigen in 12/37 dogs (32.4%) and Lewis a in the intestines of 8/37 dogs (21.6%). These antigens are derived from the activity of α1,3-fucosyltransferase on the type 1 precursor and H type 1 antigen, respectively. Unexpectedly, our results regarding Lewis antigen expression did not correlate with these previous data. Lewis a and b expression could not be demonstrated in the canine saliva or intestinal samples used in this study. This discrepancy might be due to the fact that the 1982 study concerned an analysis of glycolipids, while in our immunohistochemical approach, glycolipids are largely removed during the tissue-processing steps. The 1982 study did not consider expression of Lewis x and y, however, which are generated from the activity of α1,3-fucosyltransferase on the type 2 precursor and H type 2 antigen. In our sample population, Lewis y was identified in 12/26 dogs (46.2%), which is comparable to the level of Lewis b expression reported in the primary study. Though the precursor antigen is still questionable, these results confirm that Lewis antigen expression is present and polymorphic in the canine population. More extensive phenotyping studies are required to solidify the results for all types of HBGA expression, but in terms of the ability of CNV to recognize HBGAs expressed in different dogs, all the dogs in this study would be potentially susceptible to infection. This conclusion is supported by recent work from our laboratory, which identified unexpectedly high seroprevalence to CNV in the United Kingdom dog population, indicating that the majority of dogs studied were susceptible to infection (18).

The confirmation that A antigen and Lewis antigen can be expressed in dogs and that the expression is polymorphic raises questions regarding the possible biological role of these HBGAs in the canine population. It has previously been suggested that variation in cell surface carbohydrate expression could be an evolutionary strategy to avoid having pathogens affect an entire population equally (13, 41). The identification of CNV as a pathogen that can bind to A antigen and Lewis antigen adds support to this theory for the dog population. It is hypothesized that dogs expressing these antigens will have altered susceptibility to CNV infection compared to dogs negative for these antigens. Though natural CNV in dogs is not believed to produce high mortality rates (20, 42, 43), it is theorized that a survival advantage may be conferred by specific phenotypes, as discussed below. However, confirmation or refutation of this theory requires a much greater understanding of the pathology induced by CNV, as well as a much wider appreciation of carbohydrate polymorphism in dogs.

The range of HBGAs identified as being attachment factors for CNV was found to be most similar to those of GI.1 Norwalk virus. Norwalk was shown to attach to H type 1 by Marionneau et al. (11), and subsequent studies identified Lewis b and the A antigen as additional attachment factors (30, 44, 45). In addition, Norwalk virus has been shown to bind to H type 2 and H type 3 structures (11, 30), although the binding has weaker affinity than that of H type 1 (46). This binding pattern is remarkably similar to that identified for CNV, and although there appear to be minor variations between the CNV strains and the degree to which HBGAs are recognized, the overall specificities are very similar. The results from the synthetic oligosaccharide experiments at 4°C and the FACS data from the glycosyltransferase-transfected CHO cell studies have demonstrated that CNV can recognize H type 2 and H type 3 structures, albeit with reduced binding ability in comparison with H type 1. Whereas CNV and Norwalk virus HBGA recognition patterns are highly comparable, HBGA-binding patterns of other human noroviruses are quite distinct from that of Norwalk virus and thus CNV. For example, GII.5 strains recognize the A and B antigens but not the H antigen, GII.9 strains can recognize the Lewis a antigen, and GII.4 strains recognize A/B/H and Lewis antigens (45, 46).

The likely ability of CNV to infect all dogs is in contrast to the proportion of the human population that are susceptible to GI.1 Norwalk virus. As with CNV, Norwalk virus cannot bind to human tissues or saliva that do not express HBGAs on epithelial cells (nonsecretors) (11), and binding to B-positive saliva or red blood cells is significantly less than for A- or O-positive samples (15, 30). Consequently, nonsecretors are not susceptible to Norwalk virus and B-positive humans have a reduced risk of infection (15, 47). Between 10% and 40% of the human population are B positive, although this varies geographically (48, 49), and 20% of Caucasians are nonsecretors (11). It has previously been suggested that noroviruses were initially introduced into humans from nonhuman hosts using HBGAs as a common niche (35). The similarities identified between HBGA recognition of GI Norwalk virus and CNV are marked. We have established that CNV appears well adapted to a secretor-positive, B antigen-negative population. Animal species other than dogs also exhibit a similar spectrum of carbohydrate expression; both cattle and pigs do not express B antigen (9, 50), and no nonsecretor pigs were conclusively identified in a recent study (51). It is interesting to speculate that GI.1 may have arisen from a norovirus adapted to a B-negative animal species, such as dogs, which would explain why it lacks this HBGA specificity. GI.1 infections are now fairly uncommon in the human population, with GII.4 human norovirus strains dominant globally. This is believed to be due in part to the ability of GII.4 to bind to a wider range of HBGAs than any other human strain (52). GII.4 noroviruses are thus able to recognize a greater proportion of the human population, outcompeting the norovirus strains with a narrower host range, such as Norwalk virus (53).

The canine saliva binding studies demonstrated that CNV VLPs do not bind to all canine samples with equal abilities. This is despite all dogs expressing H antigen, a primary determinant of CNV binding, at similar levels. Therefore, our data suggest that additional factors contribute to the ability of CNV to bind to canine tissues. This in turn suggests that variability in susceptibility to infection exists in the population. It is suspected that this might be comparable to the binding and susceptibility patterns identified for RHDV in rabbits. Like CNV, RHDV is able to bind to HBGAs and *in vitro* has been shown to be able to recognize tissue samples from any rabbit regardless of phenotype. In clinical studies, however, some rabbit phenotypes are significantly more likely to become infected than others. It has been demonstrated that this is viral dose dependent. Certain rabbit phenotypes are susceptible to very low titers of RHDV, whereas other rabbit phenotypes become infected only if exposed to very high (and thus clinically rare) viral loads (27). It is predicted that a similar situation might occur during CNV infection outbreaks. Our data begin to suggest that A antigen- and Lewis antigen-positive dogs are more likely to become infected than A-negative dogs, and we speculate that unless all phenotypes are exposed to high levels of virus, the A antigen- and Lewis antigen-positive dogs are more susceptible. In our study population of dogs, 6/26 (23%) were A antigen and Lewis antigen negative. Recent CNV serology data demonstrated that approximately 40% of the dogs in a United Kingdom group of 173 dogs had not seroconverted, and we hypothesize that this may in part be due to an A antigen- and Lewis antigen-negative phenotype. Testing this hypothesis requires phenotyping dogs identified with natural CNV infection, which we aim to address in future work.

Given the close genetic relatedness of CNV to some human norovirus strains, it has previously been proposed that CNV could be a zoonotic agent. In a region where CNV was known to be circulating, serological analysis of veterinarians identified antibodies to a CNV genogroup VI strain (54). The finding that CNV recognizes HBGAs common to both dogs and humans provides some support for this suggestion. The binding experiments conducted as part of this work using human saliva samples confirm that CNV can attach to carbohydrates present on human cells and in secretions. This demonstrates that the initial step required for CNV entry into human cells is present. However, it should be noted that RHDV can bind to HBGAs (H type 2, A antigen, and B antigen) (10), and yet there is no evidence that RDHV can infect humans. HBGA binding may be an initial step in calicivirus-host interaction, but a subsequent host-restrictive step(s) must be necessary for RHDV infection, and potentially CNV infection.

In summary, this study has identified HBGAs as the carbohydrate attachment factor for CNV strains in both genogroup IV and genogroup VI. We hypothesize that HBGAs are attachment factors for other noncanine strains in these genogroups, although this requires further confirmation. Identification of a third and fourth genogroup of noroviruses that recognize HBGAs raises key questions regarding the evolutionary ancestors of these viruses. Continued investigations into the receptors/coreceptors for the currently uncharacterized norovirus strains is essential to understand the past and possible future evolutionary directions of this important viral genus.
